# The Influence of Metacognitive Strategies on the Improvement of Reaction Inhibition Processes in Children with ADHD

**DOI:** 10.3390/ijerph18030878

**Published:** 2021-01-20

**Authors:** Natalia Kajka, Agnieszka Kulik

**Affiliations:** Department of Psychotherapy and Health Psychology, The John Paul II Catholic University of Lublin, 20-950 Lublin, Poland; agnieszka.kulik@kul.pl

**Keywords:** ADHD, reaction inhibition processes, metacognitive training, Mind Maps, Sketchnoting

## Abstract

Background: Low response inhibition underlies attention disorders and hyperactivity. The aim of this study is to check whether these processes will be strengthened by three months of training with metacognitive strategies. Methodology: Forty-five schoolchildren took part in an experimental study (*M* = 10.41; *SD* = 1.42). Each child had been diagnosed with attention deficit hyperactivity disorder (ADHD). The participants were randomly assigned into three groups: the first group was tested for the effect of Mind Maps; the second group, for the effect of Sketchnoting, while the third group was assigned the role of a Control group. All of the groups were examined with the Loud Subtraction 7 test (LS7T) with a distractor before and after the training. Results: Analysis with the Wilcoxon test showed that children with ADHD made significantly fewer errors in the LS7 Test in the second measurement in the Mind Maps group (*M*1 = 7.45; *SD*1 = 4.07; *M*2 = 5.76; *SD*2 = 4.68; *p* = 0.02). In the remaining groups, there were no statistically significant differences in the average number of errors made. Conclusions: Mind Maps are an effective metacognitive strategy. Regular use of this method strengthens the inhibition of children with ADHD in this study. It can complement the existing forms of support for the child.

## 1. Introduction

Response inhibition processes are often a common feature of the most popular concepts explaining the mechanisms of attention deficit hyperactivity disorder (ADHD) [1,2,3,4,5,6,7]. Barkley indicated that impaired inhibition plays a central role in his approach and explained the occurrence of symptoms in children with this diagnosis [3,4,6]. He understood them as complex skills relating to both cognitive and behavioural activities [3]. They consist of three interrelated processes: the inhibition of the first automatic response, the ability to interrupt an ongoing activity, and the ability to continue the activity despite present distractors [1,3,7]. This means that proper inhibition allows a specific behaviour or thought to be delayed [1,7,8]. In turn, impaired inhibition will manifest itself in behaviours such as low resistance to temptation, inability to defer gratification, or inability to execute the command correctly during simultaneous distracting stimuli. Therefore, the treatment of children with ADHD should focus on enhancing the inhibition of the reaction.

There are various methods of support available for people with ADHD [9,10]. It is observed, however, that these methods only work for as long as they are applied. Barkley dared to refer to such measures as palliative [11]. This leads to the search for effective forms of work and theories that would answer the question of the cause and mechanisms of ADHD as unequivocally as possible. Barkley predicts that strengthening the response inhibition processes may contribute to a child’s better control of behaviour [3,4,7]. Some researchers also suggest that executive functions (including inhibitory processes) can be trained and improved [12]. This is important because early effective support for a child may reduce the risk of aggravation of their disorder [13]. In 2014, Barkley, together with other researchers, he proposed that children should be taught specific strategies that they can put into practice. This will allow them to improve their time management and learning processes [7]. Such actions are characteristic of metacognitive training. It is a short-term form of work originating from the cognitive-behavioural therapy (CBT). The term metacognitive is defined as knowledge about cognitive processes. Such awareness allows for the modification of the patient’s maladaptive beliefs and behaviour [14]. The literature on the subject lists various tasks that can help children. These include techniques for identifying the goal for which the child is aiming, as well as the development of effective strategies to achieve the desired goals [15]. In addition, children are taught to identify alternative strategies that will allow them to achieve the same goal in various ways [15,16]. Especially in this case, strategies of effective self-management in time (Mind Maps, organisers, calendars, reminders) or effective learning strategies (methods of visual thinking, adjusted to the individual thinking pattern) are recommended [17,18,19,20,21]. The whole task is to overcome the difficulties at the level of the patient’s self-awareness, i.e., to help them realise what resources they have at their disposal and what they should still be working on. Current reports from studies on the use of such training confirm the reduction in ADHD symptoms, better inhibition control, visual-motor coordination, or the enhancement of working memory. Additionally, there is also an increase in the children’s academic success [17,19,22,23].

It has been noticed that both Mind Maps and Sketchnoting improve the processes of planning, remembering, focus and self-regulation [17,18,19,24,25,26]. These techniques are most often used in education among healthy children, although some researchers recommend that these tools are worth promoting among children with special educational needs, including children with ADHD [17,18,19,23]. Brain neuroimaging studies show that their use requires a person to be strongly involved at many levels, which correlates with higher brain activity (higher cognitive effort) [22]. When learning by visual methods, the occipital lobe is usually active. It is responsible for encoding and visual object recognition. Some researchers suggest that the brain of a child with ADHD is even adapted to learning with visual methods [27,28]. This is explained by the brain’s neuroplasticity and the fact that people with ADHD show the ability to compensate for difficulties in performing cognitive tasks by activating the visual-spatial and motor processing areas in the brain [27,28]. Therefore, the use of visual tools plays an important role in the cognitive processes of people with ADHD.

**Figure 1:**
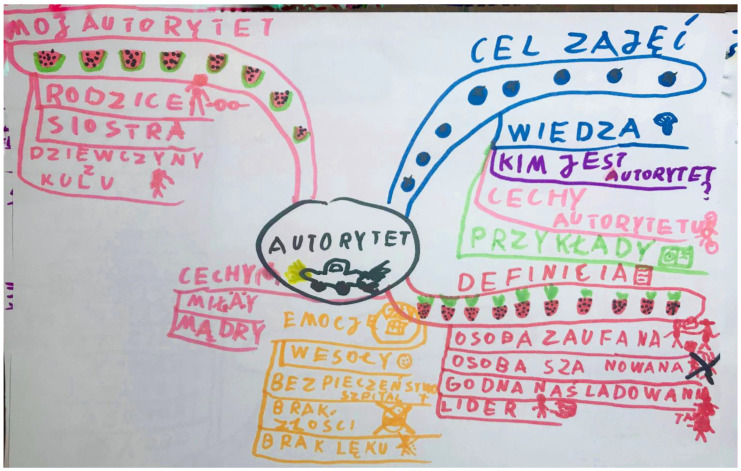
Mind Map entitled “Role model” drawn by children with attention deficit hyperactivity disorder (ADHD).

Mind Maps are a visual–written form of a note. They consist of structuring and selecting the most important information that the child operates with (Figure 1) [24].

**Figure 2:**
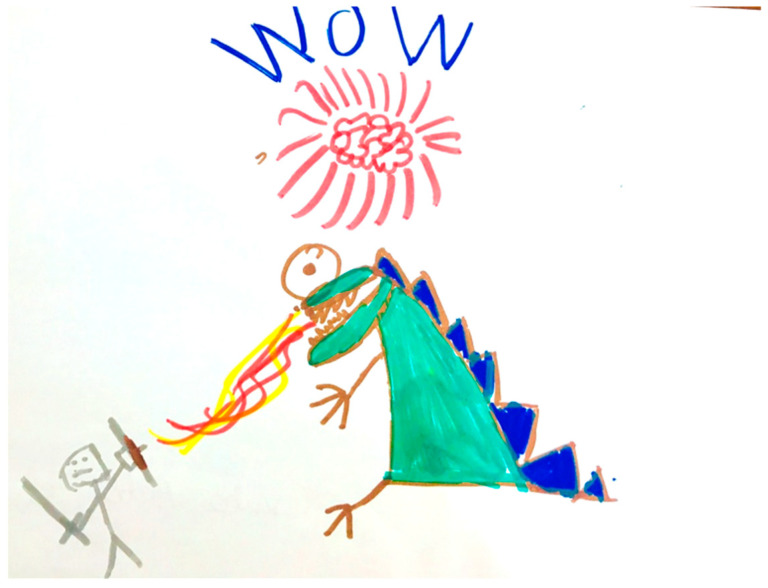
Sketchnote entitled “Role model” drawn by children with ADHD.

Sketchnoting, in turn, (Figure 2) is a visual note that is predominantly based on images. The child imagines key words important in the learning process and transforms them into a specific image. It can be supplemented with arrows, boxes, figures, and charts [25,26]. However, sketchnotes do not have a structure as orderly as Mind Maps.

To date, there are no studies available on how metacognitive stimulation using separate visual strategies could improve the response inhibition processes in children with ADHD. Therefore, the aim of this study will be to determine whether the application of selected metacognitive strategies, including Mind Maps and Sketchnoting, will have the effect of strengthening inhibition in children with ADHD.

## 2. Materials and Methods

### 2.1. Description of the Experimental Procedure

The experimental study involved 45 children aged 9–12 years (*M* = 10.41; *SD* = 1.42) diagnosed with attention deficit hyperactivity disorder with a combined presentation. Each child who joined the project had an ADHD diagnosis, which was confirmed by medical and psychological documentation. The children had no comorbidities (except for allergies). The medications taken by children were divided (based on the medical leaflet) between those influencing cognitive processes positively, negatively and those that were indifferent to these processes (Table 1). In the group assigned to Mind Maps, 33.33% of respondents were taking medication. Three children were taking medication that had a positive effect on improving concentration. One child was taking medication that had a negative effect on attention, and one person was taking a neutral medication. In the Sketchnoting and Control groups, 26.7% of children were taking medication. Three people took drugs affecting the ability to concentrate, and one person took drugs belonging to the indifferent group. Detailed characteristics of the people taking part in the study are presented in Table 1.

The subjects were randomly assigned to three groups: (1) with Mind Map stimulation, (2) with Sketchnoting, and (3) the Control group. Parents whose children were included in the experimental groups were informed that their children took part in metacognitive training aimed at strengthening executive functions and reducing ADHD symptoms. They were informed that in the classroom, the children would learn one of two strategies used in such training. In turn, the parents of children from the Control group received information that they were waiting in the queue for their children’s participation in training for about 3 months and after that time they would be invited by the researcher to re-test and train (which is not the subject of these studies). It is worth noting that the reported children were assigned a number on a current basis and then, using a number generator, they were randomly assigned to the Mind Map, Sketchnoting or Control group. Participants of the experimental groups had 25 individual thematic classes, twice a week, for a period of three months. Children from the Control group did not undergo metacognitive training. Each participant was examined twice, at the beginning and at the end of the training (experimental groups), and three months after the first measurement (Control group). That was the duration of the training. Training with the use of two metacognitive strategies had a clearly defined goal and course of each meeting. It was based on the Kolb series, so the classes began with a short introductory exercise, which allowed the children to reflect, which was supplemented with the necessary theory by trainers. The last step was closed with the exercise that gave the children the opportunity to perform it in accordance with the acquired knowledge and skills. At each stage, the children drew fragments of Mind Maps or sketchnotes in accordance with the topic of subject classes. Classes were conducted individually with each child by three trainers in 25 thematic meetings: (1) detective, (2) Mind Map/drawing of my life, (3) principles of Mind Map/sketchnote, (4) principles of classes, (5) senses, (6) wonders of the world, (7) my dream day, (8) self-presentation training, (9) friendship, (10) animals, (11) weather phenomena, (12) cosmos, (13) strange tools, (14) fairy tales, (15) board game, (16) illusions, (17) role model, (18) sports, (19) holidays, (20) water experiments, (21) competitions, (22) food, (23) fashion, (24) explorers, (25) summary. Each of the trainers followed the previously written procedure. It is worth emphasizing that the research team consisted of 100 academic students, who were systematically rotated during classes with children, so as to minimise the impact of relations with trainers. An example of the procedure for creating a Mind Map for a topic “role model” is given below. the Mind Map (Figure 1) consists of five main roots. The first (in the upper right corner) is the goal of the lesson. The children write out in single words the agenda of the meeting, which was presented by the trainer. In the case of the “role model” classes, the participant wrote: *knowledge of who is role model, features role model, examples of my role models*. The second root was related to the definition of a role model, which the child created on the basis of the exercise in which he participated. According to the trainee, the *role model is a trusted person, a person who is respected, a person who is worth following, a person who is a leader*. The third root concerned the emotions evoked by the role of the model: *joy, security, lack of fear, lack of anger*. The fourth root was distinguished by the characteristics of a role model: according to the child, the role model should be *nice* and *wise*. The last root presents the roles of the participant models—these are: *parents, sister, and girls from the Catholic University of Lublin (CUL)*, who are the trainers conducting the training. In the case of children who participated in the Sketchnoting group, the lesson procedure based on the Kolb’s cycle was the same. The only difference was drawing (possibly without using words). In the example of Figure 2, the participant drew a sketchnote that symbolically combined the entries recorded on Mind Maps (children, under the influence of the same exercises, came to similar conclusions). In the research participant’s concept, a knight with a shield is the embodiment of a role model (brave, strong, worth imitating). The knight finally defeats the dragon (personification of problems) with knowledge, wisdom (drawing of the brain). It arouses general admiration and the will to imitate (exclamation “Wow”).

### 2.2. Description of Research Tools

The literature on the subject indicates that the ability to inhibit the response is most often measured in children with ADHD with the following tools: Go/No-Go, Stop Signal Task, the Tower of London Test, Continuous Performance Task (CPT), Stroop Test, Stop Signal Task, the Wisconsin Card Sorting Test (WCST) or the Labyrinth Test [1,2,10,11]. Unfortunately, these tools mostly examine several executive functions simultaneously [1,11,12,13,14]. Therefore, in this study, we decided to use the Loud Subtraction 7 Test (LS7T) with an auditory distractor [29]. This test belongs to the PU-1 Cognitive Functional Diagnostic Battery, whose theoretical basis is largely based on Barkley’s concept of the response inhibition deficit [3,29]. LS7T is designed to assess the inhibition of external distractor interactions and the inhibition of access to irrelevant information [29]. In scientific research, 7-back subtraction down is used, for example when studying cognitive-motor interference [30]. LS7T tests both the inhibition of the dominant response as well as interference. Such understanding of these processes is consistent both with Barkley’s suggestion and with the results of research of other authors [3,5,31]. LS7T is based on the mechanisms of other known tests (e.g., the Stroop test or experimental trials applied in research) [30]. Despite the fact that the factor analysis of this tool showed that this test was a significant fit in the attention-related model in which the components were: orientation, selectivity and inhibition. This test does not activate the working memory, as is the case with classic n-back tasks and other popular tools [29]. Therefore, this study concluded that this tool would be an interesting alternative to existing tools. This tool is a serial subtraction of the number 7 from 100 counting down, while listening to the weather forecast in the background as a distractor. The inhibition indicator is the number of errors in the correct result [29]. LS7T has satisfactory psychometric properties.

## 3. Results

The characteristics of the results obtained by research participants are presented below. The number of mistakes made among all surveyed children range from 0 to 14 (Table 2). The smallest number of errors was made by children from the Mind Maps group (*M* = 7.45; *SD* = 5.76), and the largest number by children from the Sketchnotes group (*M* = 10.93; *SD* = 4.10). Despite this, no statistically significant differences were identified between the study groups in the first measurement (F (2,42) = 2.126; *p* = n/i). In order to check whether there are statistically significant differences between the groups, the analysis of variance (ANOVA) with the Games–Howell post hoc test was performed in the second measurement. The results in Table 1 demonstrate significant differences in the intensity of the mean score for response inhibition process variable between the Mind Map group and the Sketchnoting group, and Mind Map group and the Control group (F (2,42) = 10.02; *p* = 0.001). Children from the Mind Map group made significantly fewer mistakes than the children in the other groups, and 32% of this progress can be attributed to the Mind Map training (ω2 = 0.32).

The results of the Wilcoxon test show that the average number of inhibition mistakes in children with ADHD decreased in the second measurement under the stimulation with Mind Maps. There was no statistically significant decrease in the number of mistakes in the remaining groups. The rank of two-series correlation coefficient was also calculated, defining the effect strength index for the Wilcoxon test (rc = 0.51). Its value indicates that children who participated in training with Mind Maps commit significantly fewer mistakes, and the quantitative power of this phenomenon is high [32].

## 4. Discussion

The benefits of regular use of Mind Maps described in the literature were most often based on the opinion of practitioners (teachers, psychologists, educators, etc.) and reports from research conducted on a group of healthy students [17,18,24,33]. Similar characteristics apply to research related to the use of Sketchnoting. There are articles using visual methods in metacognitive training where children with ADHD constituted the research group, but it is difficult to determine which specific strategies improve the measured executive functions. This is because the researchers conducted various cognitive–behavioural interactions simultaneously. The data indicate an overall improvement in working memory, emotional regulation, planning and inhibition, and, moreover, parents have often been included in the support plan [22,23,34]. The aim of this study was to check whether selected specific metacognitive stimulations would lead to an improvement in response inhibition processes, which, according to many authors, are considered to be the key pathomechanism of ADHD [1,2,3,4,5]. The analyses carried out in this study show that metacognitive training with the use of the Mind Map was the only one that significantly improved this variable in the subjects. There were no statistically significant differences between the measurements in the Sketchnoting group and the Control group.

Inhibition is assumed to be a complex construct and combines both cognitive and behavioural inhibition [3,35]. Mind mapping as a method also integrates these complex processes. According to the researchers, who carried out inhibition control training for people who were supposed to refrain from responding to a stimulus, two mechanisms were noted which had different effects. These include bottom-up inhibition and top-down inhibition. If the association between stimulus and response is the same as the stimulus, then more automatic (non-reflective) forms of inhibition in the participants are involved. On the other hand, when the association of a stimulus with a reaction is more diverse, it requires greater involvement from the participant [36]. Metacognitive training provided the children with an appropriate framework of repeatability and predictability of training (each meeting had its clearly defined goal, course and working methods), as well as diversity in the exercises and topics covered, simultaneously training both inhibitory mechanisms. Additionally, the Mind Map group aimed to organise the thoughts and associations which the children had during the training, before they were marked on the map. This is how the participants created the appropriate structure for their associations. The children in the Sketchnoting group did not have such a structure. They drew their associations freely, anywhere on the page. Thus, when the children in the Mind Map group took their notes, they had more control over the material they produced. They also had a growing awareness of how certain difficulties could be resolved.

## 5. Conclusions

The present study showed that metacognitive training using Mind Maps improves the ability to inhibit the response in children with ADHD, who were involved in this experiment. These processes are a complex construct and so far in the literature they have been studied according to various paradigms using tools that measured many executive functions simultaneously. Such an approach makes it difficult to determine what is really improving, as different authors operationalise the studied variable differently. This study uses an experimental trial, only measuring inhibition (based on cognitive and behavioural mechanisms). The research was conducted on Polish children, and LS7T has Polish standardisation. Following the current scientific research reports, there is a growing importance of alternative methods of assistance and a need for empirical research to enrich the range of practical tools for professionals working with children and young people. The obtained results can be used in the therapy, diagnosis, and education of children with ADHD. This is important because early effective support for a child may reduce the risk of aggravation of their disorder and positively affect their offspring in the future [9,12,13]. However, it should be borne in mind that only 45 children participated in this study. It would be worth conducting a similar study on a larger sample to be able to generalise its conclusions to the population of children with ADHD. However, let us these results as an encouraging premise for further research.

## Figures and Tables

**Table 1:** Descriptive characteristics of the results obtained for the participants of this study.

Variables		Mind Maps Group	Sketchnoting Group	Control Group
Age	*M*	10.45	10.26	10.51
	*SD*	0.82	1.03	0.82
	Min	9	9	9.5
	Max	12	12	12
Sex (N)	female	2	3	2
	male	13	12	13
Living (%)	V	13.3	13.3	40
	T 50	0	20	0
	C 150	6.7	6.7	0
	C 400	80	60	60
socio-economic status (%)	low	15	6.7	10
	medium	70	73.3	76.7
	high	15	20	13.3
Medicine (N)	positive	3	1	1
	negative	3	0	1
	neutral	3	0	1

Annotation: V–village; T 50—town 50,000 inhabitants; C 150—city 150,000 inhabitants; C 400—city 150,000 inhabitants.

**Table 2:** Comparison of the mean scores for the response inhibition processes variable in the first and second measuremen.

Response Inhibition Process						
Group	Mind Map		Sketchnoting		Control	
Measurement	I	II	I	II	I	II
Z	1.96		0.493		0.716	
*p*	0.02		0.31		0.23	
*M*	7.45	4.07	10.93	10.8	8.2	8.6
*SD*	5.76	4.68	4.1	3.67	4.6	4.19
Me	11	0	13	12	10	10
Minimum	0	0	0	0	0	0
Maximum	14	11	14	14	13	13
ANOVA			Measurement I		Measurement II	
			F (2.42) = 2.126 *p* = s.i.		F (2/42) = 10/02 *p* = 0/001	
			MM > R, MM > K *			

Note: * Children from the Mind Map group made significantly fewer mistakes than children from the group with drawings. s.i.: statistically insignificant.

## Data Availability

The data contained in this article has not been published in the repository but is available upon request.
